# Echocardiographic assessment of aortic regurgitation: a narrative review

**DOI:** 10.1186/s44156-023-00036-7

**Published:** 2024-01-03

**Authors:** Vasiliki Tsampasian, Kelly Victor, Sanjeev Bhattacharyya, David Oxborough, Liam Ring

**Affiliations:** 1 Norwich Medical School, University of East Anglia, Norwich, NR4 7TJ UK; 2 Cleveland Clinic London, London, UK; 3 St Bartholomew’s Hospital, Barts’ Heart Centre, London, UK; 4 Research Institute of Sports and Exercise Science and Liverpool Centre for Cardiovascular Science, Liverpool John Moores University, Liverpool, UK; 5 West Suffolk Hospital NHS Foundation Trust, Bury St Edmunds, UK

**Keywords:** Aortic regurgitation, Aortic valve disease, Transthoracic echocardiography

## Abstract

Aortic regurgitation (AR) is the third most frequently encountered valve lesion and may be caused by abnormalities of the valve cusps or the aorta. Echocardiography is instrumental in the assessment of AR as it enables the delineation of valvular morphology, the mechanism of the lesion and the grading of severity. Severe AR has a major impact on the myocardium and carries a significant risk of morbidity and mortality if left untreated. Established and novel echocardiographic methods, such as global longitudinal strain and three-dimensional echocardiography, allow an estimation of this risk and provide invaluable information for patient management and prognosis. This narrative review summarises the epidemiology of AR, reviews current practices and recommendations with regards to the echocardiographic assessment of AR and outlines novel echocardiographic tools that may prove beneficial in patient assessment and management.

## Introduction

Aortic regurgitation (AR) may be secondary to abnormalities of the AV leaflets, the structure or geometry of the aortic root or the ascending aorta, or a combination of the two. AR may develop acutely or present as a chronic process, and results in diastolic blood flow reversal from the aorta to the left ventricle (LV) [1]. AR is the third most common native valvular heart disease behind aortic stenosis and mitral regurgitation with a prevalence of approximately 0.5% of the total population, increasing to almost 15% of individuals over the age of 65 [2, 3]. Furthermore, severe AR accounts for around 5% of all native valve intervention [4, 5]. With an ageing population, it is expected that healthcare professionals will encounter patients with AR increasingly frequently in clinical practice.

Severe AR is an important cause of morbidity and mortality. Left untreated, the risk of death is approximately one third over 10 years, and almost a half of all patients will develop heart failure [6, 7]. Even in asymptomatic patients, severe AR carries a noteworthy annual mortality risk of up to 2.2% [7, 8]. Echocardiography is central to the diagnosis and quantification of AR severity, in addition to delineating the aetiology and mechanism of valve insufficiency. Echocardiography is also key in the characterisation of important prognostic features including left ventricular (LV) dimensions and function, which may influence patient management. This narrative review summarises epidemiology and aetiology of AR, the evidence-base regarding echocardiographic assessment of aortic insufficiency, and novel echocardiographic tools that may prove beneficial in patient assessment and management.

## Aetiology of aortic regurgitation

AR may present and/or develop acutely or gradually and is caused by malcoaptation or malapposition of the AV cusps. This may be a result of abnormalities of the AV cusps and/or their supporting structures, including the AV annulus, the aortic root and the ascending aorta [9]. In Western countries, degenerative AV disease is the most common cause of AR, accounting for approximately half of the total cases [4]. Degenerative AV disease is more frequently encountered in the form of focal calcific deposits or diffuse fibrous thickening causing abnormal coaptation, although, rarely, myxomatous degeneration of the aortic cusps may also account for AR secondary to cusp thickening and/or prolapse [10]. Apart from myxomatous degeneration, aortic valve prolapse itself accounts for approximately 1.2% of all diagnosed AR lesions and may be encountered in patients with bicuspid AV and patients with aortic root disease, such as dissection or dilatation [11]. Rheumatic fever is the leading cause of AR in developing countries, and despite widespread use of antibiotics remains a notable cause of AR in Western countries alongside bicuspid aortic valve disease and infective endocarditis [4].

AR may also be a result of distortion of the structures that support the cusps and loss of support from the annulus, root and aorta. Minor dilatation of the ascending aorta occurs with ageing, a process mediated by cystic medial degeneration that weakens the aortic wall [12, 13]. However, this physiological process is commonly accelerated by the presence of hypertension, which leads to increased wall stress [12, 14]. Atherosclerosis may result in dilatation of the aorta, although this process is usually reserved to the descending rather than the ascending aorta [12]. A dilated ascending aorta is frequently seen to co-exist in patients with bicuspid AV and connective tissue disorders [12, 15]. Less commonly, AR may be caused by aortic dilatation and aortic root aneurysms associated with inflammatory changes in the aortic wall secondary to large vessel vasculitis and rheumatic diseases [16]. Rarely, congenital ventricular septal defects (VSDs), especially perimembranous or subarterial types of VSD, may lead to aortic valve prolapse and regurgitation as a result of loss of cusp structural support and the Venturi effect [17, 18]. Approximately half of subarterial VSDs with associated AV prolapse are complicated by AR therefore preventative early surgery is recommended, whilst for perimembranous VSDs with concomitant AV prolapse, surgery is recommended if more than trivial AR develops [18, 19].

Table 1 summarises the causes of acute and chronic aortic regurgitation.

**Table 1:** Causes of acute and chronic aortic regurgitation

Causes of chronic aortic regurgitation	
Valve abnormalities	Abnormalities of the aorta
**Congenital**	
Unicuspid/bicuspid/quadricuspid aortic valve	Aortic dilatation associated with bicuspid aortic valve
Ventricular septal defect	Annulo-aortic ectasia
Connective tissue disease • Marfan syndrome • Ehlers-Danlos syndrome • Loeys-Dietz syndrome	Connective tissue disease • Marfan syndrome • Ehlers-Danlos syndrome • Loeys-Dietz syndrome • Osteogenesis imperfecta
**Acquired**	
Degenerative • Calcific valve disease • Myxomatous valve disease	Degenerative • Systemic hypertension • Atherosclerosis
Inflammatory • Rheumatic heart disease • Radiation-induced valve disease • Toxin-induced valve disease	Inflammatory • Rheumatic diseases • Ankylosing spondylitis • Reactive arthritis • Vasculitis • Giant cell arteritis • Takayasu arteritis • Behcet’s disease • Psoriatic arthritis • Reactive arthritis
	Infectious • Syphilis

Causes of acute aortic regurgitation	
Valve abnormalities	Abnormalities of the aorta
Infective endocarditis	Aortic dissection
Traumatic injury	Traumatic injury

## Echocardiographic assessment of aortic regurgitation

### Mechanism and classification of AR

Originally designed for the mitral valve, Carpentier’s classification has been adapted for use in the assessment of AR mechanism, whereby the lesion is classified according to cusp morphology and motion [20, 21]. Type I includes AR with *normal* cusp motion, where the insufficiency is secondary to aortic root dilatation or cusp perforation; Type II AR refers to *excessive* cusp motion including aortic cusp prolapse; those with *restricted* cusp motion are grouped in type III [21] (Table 2). The functional classification is an invaluable tool that helps clinicians systematically evaluate the valve behaviour and may influence the type of intervention chosen for the valve [22, 23]. It also carries prognostic value both in terms of valve repairability and of long-term outcomes: Type III AR is associated with poorer long-term outcomes after valve sparing surgery and a higher risk of recurrent AR post valve repair [23–25]. Cusp perforation/fenestration is another important phenotype that has important implications for the choice of surgical treatment, as it has less favourable outcomes when treated with valve repair [25, 26].

**Table 2:** Anatomical classification of AR lesions according to cusp motion

Anatomical classification of AR lesions			
Type	Dysfunction	Regurgitant jet	Echocardiographic findings
I	Normal leaflet motion	Central or eccentric jet	Ia: Sinotubular junction and ascending aorta dilatation Ib: Sinuses of Valsalva and sinotubular junction dilatation Ic: Annulus dilatation Id: Cusp perforation or cusp fenestration without a primary functional aortic annular lesion
IIa	Excessive leaflet motion due to cusp prolapse	Eccentric jet	• Flail cusp • Partial cusp prolapse • Whole cusp prolapse
IIb	Free edge fenestration	Eccentric jet	Mobile fibrous strands attached to cusp near its commissure
III	Restrictive leaflet motion	Central or eccentric jet	• Thickened cusps with restricted cusp motion • Extensive calcification/calcific deposits

The degree of AV calcification may influence clinical decision making, and a grading system has been proposed: no calcification is classed as grade 1; small calcification spots (grade 2); larger calcification spots interfering with cusp motion is grade 3; extensive calcification causing restricted cusp motion is grade 4 [20]. Valve sparing or valve repair surgery is not recommended in cases with moderate or extensive cusp calcification (grades 3 & 4), due to the substantial risk of recurrence of significant AR post valvuloplasty [25, 27].

Three-dimensional (3D) echocardiography may provide additional useful information, by enabling the reconstruction, visualisation and assessment of the morphology of the AV without the geometrical assumptions involved in 2D echocardiography [28, 29]. Multi-plane imaging of the AV removes the uncertainty of the single cut-plane position in the parasternal view, allowing correct identification of all the aortic cusps [29]. It is also beneficial for visualisation of the AV throughout the cardiac cycle, overcoming the issue of through-plane motion [29]. However, 3D echocardiographic assessment of the AV is challenging and often suboptimal in cases of significant AV calcification or in patients with poor acoustic windows [28, 29].

### Severity of AR

Doppler assessment including colour flow and continue wave (CW) Doppler allow detailed assessment and visualisation of the aortic regurgitant jet and its components including the flow convergence zone, the vena contracta (VC) and the jet area [21, 30]. Assessment of these characteristics constitute the primary method of evaluation of the severity of AR [20, 21].

A simple visual assessment of the CW signal density may provide a general idea of the severity of the AR; a denser CW Doppler signal indicating more regurgitant flow and a faint signal suggesting mild regurgitation. Beam alignment is an important issue when using this method, with eccentric jets resulting in faint signals because of the Doppler error stemming from the large angle of insonation. Importantly, both moderate and severe AR result in dense CW traces; ultimately, due to the these limitations, CW signal density is not recommended to be used to quantify AR [30].

A small study demonstrated that jet width, defined as the ratio of the jet diameter divided by the Left Ventricular Outflow Tract (LVOT) diameter, correlated well with the angiographically obtained grade of AR severity: a ratio of ≥ 65% being consistent with severe AR [31]. However, very few patients were included in this study, none of whom had congenital AV disease or AR type II, limiting its value in such circumstances [31]. Subsequent work has questioned the usefulness of this parameter, which has less physiological significance than the VC, even when normalising for the LVOT diameter [32]. As such, current guidelines advocate the use of jet width as part of a multiparametric assessment of AR [21, 30]. Jet length and jet area are very much dependent on LV compliance and diastolic pressure, and do not reflect the severity of the AR; accordingly they are not recommended for use [33].

Interpretation of colour Doppler is challenging in acute severe AR. In such cases, LV diastolic pressure rises rapidly as the non-compliant LV fills with blood from both the aorta and the left atrium during diastole [20, 21, 33]. This results in the AR jet being of shorter duration and lower velocity, becoming therefore difficult to detect with colour flow Doppler only. In these cases, other echocardiographic methods should be used for the assessment of the AR severity [21, 30].

Pressure half-time (PHT) is a technique in which the rate of deceleration of the regurgitant blood flow can be measured from the CW Doppler of the AR. As AR becomes more severe, LV end-diastolic pressure increases and end-diastolic aortic pressure decreases, resulting in a smaller late diastolic gradient and therefore shorter pressure half-time [36]. Early work demonstrated that a PHT of 400ms can reliably identify important regurgitation, and angiographic grade 4 + AR correlates with a PHT of approximately 200ms [35]. Multiple subsequent reports confirm that mild AR demonstrates significantly longer PHT compared to moderate to severe AR [36–38]. There are several important drawbacks of this technique. First, it is challenging in eccentric jets in which optimised alignment of the US beam is often not possible. Secondly, correlation between the pressure half-time and severity of AR is poor in mild or moderate AR, but better in severe AR cases [34, 35, 38]. A third concern is that PHT is highly influenced by the diastolic function of the LV, with the measurement becoming unreliable in cases of impaired relaxation and/or compliance and significant co-existent LVH [39]. Finally, changes in the diastolic blood pressure secondary to medications (i.e., vasodilators) can also affect the gradient between the aorta and the LV, rendering the method less useful in patients on those medications [40]. Acknowledging these limitations, the PHT is recommended to be used as a supplementary method of assessment and grading of the AR should not rely solely on this [21, 30].

In mild AR, early flow reversal may be seen in the proximal descending thoracic aorta. As the severity of AR progresses, the duration of flow reversal extends through diastole with the reversal velocity of the blood increasing [30]. In a small study, holodiastolic flow reversal with end-diastolic velocity of ≥ 20cm/s was found to be a marker of severe AR and correlated well with a regurgitant fraction (RF) of ≥ 40% with a sensitivity and specificity of 88% and 96% respectively [30, 41]. MRI studies have confirmed the highly specific nature of this finding for severe AR [42, 43]. Colour-coded M-mode may help in the assessment of the timing of the flow signal in relation to the cardiac cycle [30]. Holodiastolic flow reversal in the abdominal aorta is also a highly specific marker of severe AR, but with moderate sensitivity [44, 45]. This sign is also commonly found in patients with congenital heart disease and aorto-pulmonary shunt, therefore, its presence on these occasions is not highly specific for severe AR [46, 47].

The VC represents the smallest flow diameter of the regurgitant jet going through the AV, and provides a surrogate for the effective regurgitant orifice area (EROA) and an indicator of the AR severity [30]. A number of studies have reported that a VC of > 5mm correlates with severe AR with a sensitivity up to 95% and specificity between 80 and 90%, making it an excellent tool in the identification of severe AR [32, 48]. The main limitations of VC include the assumption of a circular regurgitant orifice, which is often not the case. Additionally, there are no studies investigating the accuracy or prognostic role of VC in the context of multiple AR jets: in such cases guidelines advocate that the VC of the largest jet should be reported, acknowledging that this will necessarily underestimate overall severity of AR [30]. This is an important limitation for clinical use.

Although qualitative assessment of AR is used more frequently in echocardiographic practice, quantitative measures provide the clinician with prognostic information which may inform management. The proximal isovelocity surface area (PISA) method directly assesses the EROA and can be used to the derive the regurgitant volume (RV). The ratio of forward flow or stroke volume to RV can be used to determine the regurgitant fraction (RF). An EROA ≥ 0.30cm^2^, regurgitant volume ≥ 60mls and regurgitant fraction > 50% all indicate severe AR [20].

In a study of over 250 asymptomatic patients with chronic severe AR, EROA and RV were shown to be independent predictors of 10-year survival and freedom from surgery from AR [8]. In a smaller analysis, integrated assessment and quantification of AR severity closely correlated with the clinical endpoint of AV surgery [49].

Despite the prognostic value of these tools, the PISA method can be challenging with substantial cusp thickening and/or calcification influencing the visible convergence zone. Additionally, the EROA appears to be significantly underestimated when there is an obtuse flow convergence zone angle (> 220°) [50]. In the presence of eccentric jets, PISA tends to underestimate AR severity although these limitations can be overcome when the assessment is performed from the left parasternal instead of the apical window [51].

The calculation of the regurgitant volume requires the VTI obtained from the CW Doppler envelope of the regurgitation, which may be challenging to obtain in eccentric jets when alignment of the US beam is difficult. Regurgitant volume can also be derived by calculating the difference in the stroke volume through the LVOT and the mitral valve inflow. This method is time-consuming, can only be applied if there is no significant co-existent mitral or pulmonary regurgitation, and is subject to significant inter-observer variability and errors in linear dimensions that may substantially impact on the final result [20].

In cases of acute AR or when LV is impaired and there is reduced LV stroke volume, both the EROA and the regurgitant volume may underestimate the severity of AR: in such circumstances, the RF may be more useful at indicating severe AR [30]. Early mitral valve closure and diastolic mitral regurgitation (MR) are important echocardiographic signs that may alter the clinical course and management of patients with acute severe AR. Premature closure of the mitral valve may be categorised as grade I (up to 50ms before the Q wave) or as grade II (up to 200ms before the Q wave) and is a specific and sensitive indicator of acute severe AR [52]. Patients with grade II early mitral valve closure usually suffer significant elevations in their LV diastolic pressure and volume which cannot be adequately compensated [52]. Therefore, their presence suggests urgent surgical intervention [52, 53]. In addition, the presence of diastolic mitral regurgitation is an independent predictor of pulmonary oedema and/or haemodynamic instability in patients with acute severe AR and therefore is another echocardiographic finding that may play an important role in patient’s management plan and prognosis [54].

In summary, a quantitative assessment of AR should be routinely performed for those patients with more than mild AR [20, 21, 30]. Additional parameters are useful if there is disagreement between these parameters and to corroborate the conclusion of quantitative assessment. Of the additional techniques, diastolic flow reversal in the descending aorta is the strongest parameter for the evaluation of the severity of AR [20]. Table 3 summarises the echocardiographic indicators of severe AR as per the American and European guidelines.

**Table 3:** Markers of severe Aortic Regurgitation (AR) according to international guidelines

Markers of severe AR		
	American College of Cardiology & American Heart Association [21, 56]	European Association of Cardiovascular Imaging & European Society of Cardiology [20, 30]
**Qualitative parameters**		
Doppler jet width	Large in central jets	Large in central jets
	Variable in eccentric jets	Variable in eccentric jets
Flow convergence zone	Large	Large
Diastolic flow reversal in descending aorta	Prominent holodiastolic reversal	Holodiastolic flow reversal (End-diastolic velocity ≥ 20 cm/s)
**Semi-quantitative parameters**		
Vena contracta width	> 0.6cm	> 0.6cm
Jet width / LVOT width	≥ 65%	≥ 65%
Pressure half time	< 200ms	< 200ms
**Quantitative parameters**		
Effective Regurgitant Orifice Area	≥ 0.30cm^2^	≥ 0.30cm^2^
Regurgitant volume	≥ 60mls	≥ 60mls
Regurgitant fraction	≥ 50%	≥ 50%

*CSA* Cross-sectional area; *LVOT* Left ventricular outflow tract; *ms* milliseconds; *mls* millilitres; cm, centimetres

An algorithmic approach and hierarchical weighting of key echocardiographic parameters may be extremely helpful when grading the severity of AR [55]. Multiparametric assessment, as recommended by the current international guidelines, is a useful approach in the evaluation of the AR severity, however it increases the risk of interobserver variability of AR assessment and leads to significant inconsistencies between the assessors. This phenomenon becomes more pronounced in the presence of discordant parameters [55]. Preferential weighting of selected echocardiographic parameters may overcome this important limitation. Using a practical algorithm based on parameters both useful and highly influential when grading the severity of AR, minimises interobserver variability and improves concordance and accuracy [55]. Each of the echocardiographic parameters in isolation may have several limitations that make the grading of AR severity challenging and problematic. A practical algorithmic approach that incorporates not only a certain number of parameters but also the significance of each parameter, can help overcome this challenge and adopt a consistent and accurate method of assessing AR severity.

### Haemodynamic consequences of AR

Chronic severe AR has important haemodynamic consequences that affect the LV size and function. Long-standing volume overload results in LV remodelling, which ultimately results in maladaptive changes to the myocardium, decline of LV function and the development of symptoms [57]. Multiple studies have shown that increased LV size and impaired systolic function are independently associated with adverse events and poor long-term survival [58–68]. AV surgery is therefore a Class I recommendation for patients with severe AR and impaired LV systolic function (LVEF ≤ 50%) or significantly dilated LV (LV end-systolic diameter > 50mm, indexed LV end-systolic diameter > 25mm/m^2^ or LV end-diastolic diameter > 65mm), even in the absence of symptoms [69].

Indexed LV end-systolic diameter (LVESDi) is an indicator of LV volume overload and systolic shortening. In a study of 1,417 patients with severe AR and minimal or no symptoms, there was a significant increase in mortality with an LVESDi > 20 mm/m^2^, a markedly lower cut-off than the guideline-recommended surgical threshold [6]. This cut-off value was confirmed by two further observational studies with a total combined population of more than 1000 patients [70, 71]. In another study of 284 patients, LVESD ≥ 45 mm was found to be an independent predictor of postsurgical mortality [72]. The LVEF threshold has also been challenged: observational studies suggest that 10-year mortality rates and adverse events are significantly higher in patients with an LVEF ≤ 55% when compared to those patients with an LVEF > 55% [63, 70]. Acknowledging the importance of these data, ESC guidelines suggest the consideration of surgery when LVESDi > 20 mm/m^2^ or LVEF < 55% as class IIb recommendation in low-risk cases [69]. Volumetric assessment of the left ventricle has also been shown to be significant, with several studies demonstrating that indexed LV end-systolic volume (LVESVi) of 45 mL/m^2^ or greater is significantly associated with an increased risk of mortality and adverse events [67, 68, 73]. In fact, there is evidence to suggest that the prognostic significance of LVESVi with mortality is stronger than that of the linear dimensions [73].

For patients with severe AR who do not meet the currently recommended criteria for surgery, regular echocardiographic monitoring is recommended, as serial changes in LV function and dimensions may identify those that are most likely to develop symptoms and need operation in the near future [56, 69, 71]. Asymptomatic patients with moderate and severe AR should have echocardiographic assessment on an annual basis, whilst those approaching the thresholds for intervention should be followed up at 3–6 monthly intervals [69, 74]. For patients with mild-to-moderate AR, echocardiographic assessment every 2–3 years is a reasonable timeline of surveillance [69, 74].

LV size and function is of high importance for patients’ post-surgical mortality and morbidity [64, 75]. Significant LV dilatation and severely reduced LV systolic function (defined as LVEF < 35%) are associated with poor postoperative short- and long-term outcomes [65, 76]. Smaller baseline indexed LV systolic and diastolic dimensions are associated with early recovery of the LV systolic function after valve surgery [77]. Furthermore, a study with 69 patients who underwent AVR for severe AR demonstrated that postoperative reverse remodelling is associated with better 10-year outcomes and survival rates [78]. Table 4 summarises the main findings of the studies that have examined the prognostic significance of LV structural and functional remodelling parameters in patients with AR.

**Table 4:** Studies that have examined the significance of left ventricular structural and functional remodelling in aortic regurgitation (in chronological order)

Study	Year	Follow-up	Study population	Main findings
Henry et al. [79]	1980	5–43 months	50 patients who underwent AVR for chronic severe AR	Pre-operative LVESD > 55mm and fractional shortening < 25% were strongly associated with high risk of post-operative heart failure or death or both
Kumpuris et al. [80]	1982	8 months	43 patients with chronic and 14 patients with acute severe AR that underwent AVR	In patients with acute AR, LV dimensions normalised after AVR. In patients with chronic AR, some had persistent post-op LV dilatation. LVESD > 50mm was predictive of irreversible cardiac dilation
Gaasch et al. [58]	1983	1–6 years	32 patients who underwent AVR for severe AR	25 patients achieved normal LVEDDi post-operatively. These patients had less symptoms and significantly better 4-year survival, compared to the 7 patients who had persistent left ventricular enlargement. Pre-operative LVESDi > 25mm/m^2^ and LVEDDi > 38mm/m^2^ are predictive markers of persistent post-operative LV enlargement
Bonow et al. [60]	1988	3–7 years	61 patients who underwent AVR for chronic severe AR	short-term and long-term improvement in left ventricular systolic function after operation is related significantly to the early reduction in left ventricular dilatation
Tornos et al. [59]	1995	10 ± 6 years	101 asymptomatic patients with chronic severe AR	LVESD > 50mm and LVEF < 60% were independent predictors of cardiac symptoms or LV dysfunction
Tarasoutchi et al. [81]	2003	10 years	75 patients with chronic severe AR	LVESD and age were the most predictive and specific, but not sensitive, indicators of symptom development. 10-year probability of developing symptoms was 58% for patients with LVEDD ≥ 70mm and 76% for patients with LVESD ≥ 50mm
Sambola et al. [82]	2008	8 ± 6 years	147 patients who underwent AVR for chronic severe AR	LVESD and LVESDi were independent predictors of mortality after surgery. In patients with low BSA (≤ 1.68mm^2^), LVESDi ≥ 25mm/m^2^ should be used as a cut-off point for surgery rather than LVESD > 50mm
Brown et al. [76]	2009	10 years	301 patients who had AVR for moderate or greater AVR	LVESDi and LVEDDi were predictors of late survival. Patients with LVESDi > 20mm/m^2^ and LVEDDi > 30mm/m^2^ had significantly worse 10-year mortality post-operatively
Cho et al. [77]	2010	6 months	171 patients who underwent AVR for chronic severe AR	Preoperative LVESDi and LVEDDi were independent predictors of the LV post-surgical recovery. The sensitivity and specificity in predicting normalisation of LV function were 88% and 92% for indexed LVESDi < 35.32 mm/m^2^ and 71% and 86% for LVEDDi < 44.42mm/m^2^
Park et al. [72]	2012	39.9 months	284 patients who underwent AVR for chronic severe AR	Preoperative LVESD ≥ 45 mm and haemoglobin level < 13.4 g/dl are independent prognostic factors of survival after aortic valve surgery in patients with chronic severe AR and normal LV EFs
Saisho et al. [83]	2015	10 & 20 years	177 patients who underwent AVR for chronic severe AR	LVESDi and cardiac index were independent predictors of LV recovery post-operatively. LVESDi > 26.7mm/m^2^ was the best cut-off value for predicting EF recovery after surgery
Zhang et al. [66]	2015	2 years	105 patients who underwent AVR for chronic severe AR	Pre-operative EF > 52% is a good predictor of successful LV recovery early after AVR
Wang et al. [63]	2016	10 years	192 asymptomatic patients with severe AR, LVEF ≥ 50% and LVEDD > 70 mm who underwent AVR	Pre-operative LVEF < 55% and LVEDD ≥ 81 mm were associated with poorer prognosis (5- and 10- year survival rates) in patients undergoing AVR
Bruno et al. [84]	2017	21 months	119 patients who underwent AVR for chronic severe AR	Long-term postoperative survival was not affected by baseline EF, but age > 70 years and NYHA class III/IV symptoms were predictive of survival. In-hospital and long-term survival was similar in patients with severe LV dysfunction and with preserved or moderately reduced LV function
Maeda et al. [75]	2019	10 ± 5 years	268 patients who underwent AVR for chronic severe AR	Long-term (10-year) survival after AVR was significantly worse in patients with LVESDi > 25mm/ m^2^ and/or LVEDD > 65mm
De Meester et al. [70]	2019	10 years	356 patients who underwent AVR for chronic severe AR	LVEF < 55% and LVESDi > 20 to 22 mm/m^2^ were markers of increased 10-year mortality. LV end-diastolic dimensions did not influence outcomes
Yang et al. [71]	2019	4.9 years	748 patients with significant AR, of whom 361 patients had AVR	LVESDi was the only LV parameter independently associated with all-cause mortality. Compared with patients having LVESDi < 20 mm/m^2^, those with LVESDi 20 to 25 mm/m^2^ (HR: 1.53; 95% CI: 1.01–2.31) and ≥ 25 mm/m^2^ (HR: 2.23; 95% CI: 1.32–3.77) had increased risk of death
Dong et al. [65]	2020	10 years	212 patients with LVEF < 50% and LVEDD ≥ 70mm who underwent AVR for chronic severe AR	In-hospital mortality was associated with preoperative age and LVEF. Patients with markedly reduced LV function (LVEF < 35%) had lower survival rates compared with other patients with moderate LV dysfunction (LVEF 36% to 50%)
Kim et al. [64]	2020	8.7 years	280 patients who underwent AVR for chronic severe AR	Patients with reduced LVEF (< 50%) had lower overall postoperative survival and cardiac mortality‐free survival rates than the preserved LVEF group at 5 and 10 years. Preoperative E/e′ was associated with postoperative improvement or normalization of LVEF and all‐cause mortality in the patients with reduced LVEF
Koga-Ikuta et al. [85]	2021	1 year	246 patients who underwent AVR for chronic severe AR	Pre-operative LVEF and LVESDi were significant predictive factors of reverse remodelling 1 year after surgery, which was associated with late outcomes
Yang et al. [67]	2021	5.4 years	492 asymptomatic patients with chronic moderate to severe and severe AR	LVEF, LVESDi and LVESVi were independently associated with mortality. A LVESVi threshold of 45 mL/m2 or greater was significantly associated with an increased mortality risk
Anand et al. [73]	2021	5.4 years	1100 patients with chronic moderate to severe and severe AR	Both LVESVi and LVESDi were associated with worse outcomes, but the association of LVESVi was stronger. LVESVi ≥ 45 mL/m^2^ was associated with worse outcomes
Iliuta et al. [86]	2022	2 years	332 patients who underwent AVR for chronic severe AR	The restrictive LV diastolic filling pattern was an independent predictor for early and medium-term postoperative mortality. Other independent predictors for increased early postoperative mortality rate include advanced age (> 75 years), LVESD > 58 mm, and comorbidities (diabetes mellitus, COPD)
Yang et al. [68]	2023	4.1 years	1259 patients with chronic moderate to severe and severe AR	All-cause and cardiovascular mortality were increased when LVEF ≤ 53%, LVESDi ≥ 22 mm/m^2^ and LVESVi ≥ of 46 mL/m^2^. Early surgery was beneficial in 3 strata of LVESDi (< 20, 20 to < 25, and ≥ 25 mm/m2) and 2 strata of LVESVi (< 46 and ≥ 46 mL/m^2^)

*AR* aortic regurgitation; *AVR* aortic valve replacement; *COPD* Chronic obstructive pulmonary disease; *LV* Left ventricular; *LVEDD* Left ventricular end-diastolic diameter; *LVEDDi* Left ventricular end-diastolic diameter indexed to body surface area; *LVEF* Left ventricular ejection fraction; *LVESD* Left ventricular end-systolic diameter; *LVESDi* Left ventricular end-systolic diameter indexed to body surface area; *LVESVi* Left ventricular end-systolic volume indexed to body surface area; *NYHA* New York Heart Association

Given its significant prognostic value, echocardiographic assessment of the LV is of paramount importance in the evaluation and management of patients with aortic regurgitation not only before but also after they have AV surgery.

### Novel echocardiographic indices

3D transthoracic echocardiography facilitates advanced assessment of the valve anatomy and severity of the regurgitation. 3D interrogation allows delineation of anatomical features of the AV and nearby structures, such as inter-commissural distance and aortic annular diameter, that may be used for preoperative planning [29, 87]. As mentioned previously, a major limitation of the VC method is the assumption of a circular regurgitant orifice. 3D colour Doppler echocardiography allows visualisation of the VC in simultaneous orthogonal views and enables an assessment of the cross-sectional area of the VC [88]. This method has been shown to correlate well with Cardiac Magnetic Resonance (CMR) imaging, aortographic and surgical grading of AR severity [43, 88]. A number of small studies have suggested differing cut-off values for 3D VC, ranging from 30mm^2^ to 60mm^2^ that correspond with severe AR [88–91]. As yet, there is no outcome data for 3D VC, therefore further validation is required before it is widely incorporated into practice.

Myocardial deformation or strain imaging may allow the identification of subclinical myocardial impairment present even with a normal LVEF. LV global longitudinal strain (GLS) is significantly reduced in patients with severe AR and otherwise normal LVEF [92, 93]. Several observational studies have demonstrated that GLS is an independent predictor of mortality in patients with severe AR [94–97]. A recently published systematic review reports that worse values of GLS are associated with poor cardiovascular outcomes [98]. Owing to marked heterogeneity between the studies in the analysis, most of which included fewer than 100 patients, no specific threshold value of GLS could be identified that may be of clinical use, but certainly further investigation is warranted in this regard. In a large observational study including over 1000 patients with chronic asymptomatic severe AR, GLS was independently associated with 5-year all-cause mortality [96]. Interestingly all deaths in this study occurred in patients who did not meet criteria for intervention according to the current guidelines. The reasons for this are not clear, but the authors suggest that reliance on conventional tools in the assessment of LV remodelling are likely inadequate in an overall assessment of cardiovascular risk [96]. GLS has a prognostic value postoperatively, with impaired GLS values both immediately following surgery and persistently after intervention being associated with increased long-term mortality [95, 99].

**Fig. 1:**
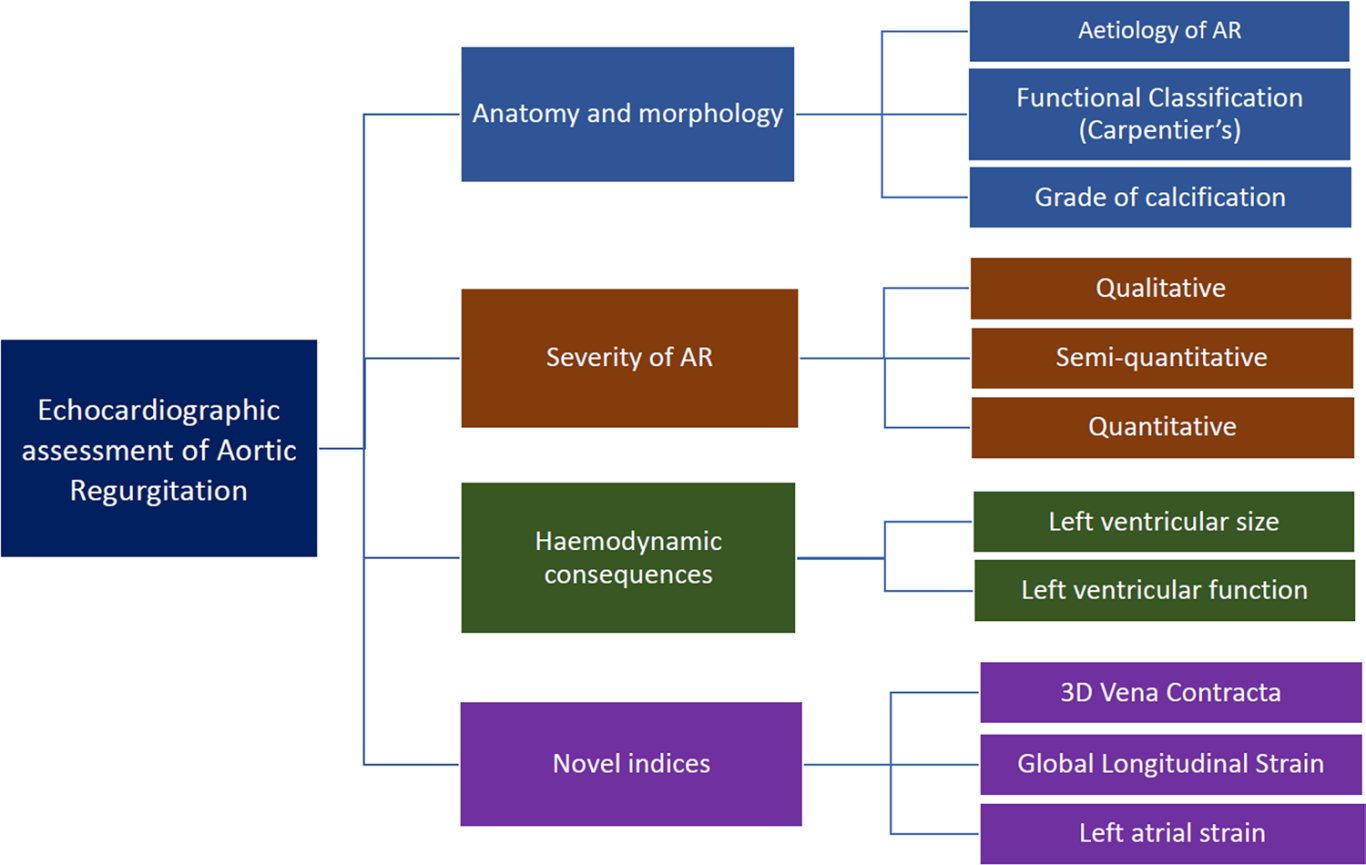
Summary of key echocardiographic parameters for the assessment of Aortic Regurgitation (AR)

Strain imaging is not limited to the LV. Left atrial (LA) reservoir strain has been documented to be impaired in patients with chronic asymptomatic severe AR, but lower values of LA strain are associated with adverse prognosis, and show promise in risk stratification of patients with severe AR [100–102]. Figure 1 provides a summary of all the parameters of significance in the echocardiographic assessment of AR (Fig. 1).

An extremely novel approach is to assess cardiac mechanics with strain-volume loops, which may allow a deeper understanding into the haemodynamic consequences of AR. Whereas LVEF and strain do not necessarily distinguish between LV remodelling in valve disease and normal controls, strain-volume loops were significantly better at identifying adverse LV remodelling compared to the conventional echocardiographic approach [103].

Table 5 provides a summary of the studies that have investigated the role of strain parameters in AR.

**Table 5:** Studies evaluating the role of strain parameters in aortic regurgitation (in chronological order)

Study	Year	Follow-up	Study population	Main findings
Marciniak et al. [93]	2009	N/A	59 patients with mild/moderate/severe AR and 22 healthy controls	Radial as well as longitudinal peak systolic strain rates were significantly decreased in patients with both moderate and severe AR compared with healthy subjects. Changes in regional LV deformation correlated inversely both with LV end-diastolic volume and with end-systolic volume
Olsen et al. [95]	2011	19 ± 8 months	64 patients with chronic severe AR	Reduced myocardial systolic strain, systolic strain rate and early diastolic strain rate by speckle-tracking echocardiography were associated with disease progression during conservative management and with impaired outcome after surgery. Conventional parameters of LV function and size (LVEF and LVEDDi) were associated with outcome after surgery but not with outcome during conservative management
Mizarienė et al. [104]	2012	N/A	26 patients with moderate AR, 34 patients with severe AR and 28 healthy controls	The LV GLS, radial strain, and LV systolic diameter were the independent predictors of LV ejection fraction in the patients with AR. LV long-axis dysfunction with an increased apical rotation was present in the patients with moderate AR, while LV radial function and systolic basal rotation were found to be reduced in more advanced disease
Ewe et al. [105]	2015	4.2 ± 3.2 years	129 patients with moderate-to-severe or severe AR and LVEF > 50%	In asymptomatic patients, impaired baseline LV GLS or circumferential strain was independently associated with the need for AVR
Lavine et al. [97]	2015	N/A	27 patients with no pathology, 87 patients with chronic AR and LVEF > 50%, 66 patients with chronic AR and LVEF < 50% and 82 patients with hypertensive heart disease	In chronic AR there is impaired longitudinal function despite preserved EF: GLS was reduced in all patients with AR compared to normal subjects, GLS was also reduced in patients with AR and LVEF > 50% compared to patients with hypertensive heart disease. GLS was well correlated with non-invasive estimated LV filling pressures and pulmonary systolic arterial pressures
Park et al. [94]	2015	5.3 years	60 patients with chronic severe AR (median follow up 64 months)	On multivariate analysis decreased LV global strain rate (measured on apical four chamber view) was proved to be an independent predictor of mortality in patients with chronic AR
Hulshof et al. [103]	2017	N/A	7 patients with severe AR, 10 patients with severe AS and 10 healthy controls	Distinct strain–volume loop characteristics were present in the 3 subgroups who showed comparable longitudinal peak strain. Early systolic strain and linear slope during systole (relationship between strain and volume) were lower in AR and AS patients compared with control subjects, with AR patients demonstrating lower values compared with AS
Verseckaite et al. [106]	2018	5 years	67 asymptomatic patients with chronic moderate or severe AR and LVEF > 50%	GLS was an independent predictor of LVEF deterioration. Probability of LVEF deterioration was significantly greater in patients with GLS
Alashi et al. [96]	2019	6.95 years	865 patients with severe AR and LVEF ≥ 50% who underwent AVR	Baseline LV-GLS value worse than -19% was associated with reduced survival. In a subgroup of patients who returned for 3- and 12-month follow-up examinations, persistently impaired LV-GLS was associated with increased mortality
Kalkan et al. [101]	2021	N/A	64 patients with mild, moderate and severe AR	This study showed that LA-Res and LA pump parameters of the patients with severe AR significantly decreased compared to those of the mild and moderate AR group
Jenner et al. [100]	2021	1 year	65 patients with severe AR who underwent AVR	Preoperative left atrial strain during the conduit phase added to LVESVi for the prediction of impaired LV functional and structural recovery after aortic valve replacement (accuracy 70%; addition of left atrial strain during the conduit phase to LVESVi p = 0.006)
Martín et al. [102]	2022	2.8 years	126 asymptomatic patients with chronic severe AR	LVEDV and E/e’ ratio were significant predictors of adverse events. Lower LA reservoir strain values (less than median of 34%) were associated with higher rates of events (hospital admission due to heart failure, cardiovascular mortality, or aortic valve surgery)

*AR* aortic regurgitation; *AS* Aortic stenosis; *GLS* Global longitudinal strain; *LV* Left ventricular; *LVEDDi* Left ventricular end-diastolic diameter indexed to body surface area; *LVEF* Left ventricular ejection fraction; *LVEDV* Left ventricular end-diastolic volume; *LVESVi* Left ventricular end-systolic volume indexed to body surface area

### Future directions

Whilst current guidance is frequently derived from an evidence-base that consists of small studies conducted more than two decades ago, the future holds promise with a series of robust studies that will hopefully complement the current data, and improve the echocardiographic assessment of AR. Larger prospective studies have already set out to answer several questions around the established and novel echocardiographic parameters used and their potential additive value in risk stratification and management of these patients.

The ‘Early Aortic Valve Surgery Versus Watchful Waiting Strategy in Severe Asymptomatic Aortic Regurgitation’ (ELEANOR) study (NCT05438862) is a prospective randomised trial investigating optimal timing of surgical intervention in asymptomatic patients with severe AR. Patients are randomised to watchful waiting approach with guideline-indicated intervention, or to early surgery. Participants undergo echocardiographic and other advanced imaging assessments at regular follow-up intervals. This study should provide insight into the prognostic value of echocardiographic parameters and how these identify patients’ clinical trajectory and adverse events.

Another upcoming study is the ‘Comparative Imaging Assessment of Valvular Heart Disease’ prospective observational study (NCT04126018) is investigating the accuracy of 2D and 3D echocardiographic methods of valvular quantification and is due to complete recruitment in 2023. Approximately 40 participants with moderate or severe valvular lesions, including AR, aortic stenosis and mitral regurgitation, will undergo 2D and 3D transthoracic echocardiographic studies. Conventional echocardiographic tools including Doppler, PISA, VC and volumetric method, will be compared to the reference standard of CMR, and will correlate with clinical outcomes.

## Conclusion

Aortic regurgitation is a common valvular heart disease with significant impact on patient mortality and morbidity. Echocardiography is a simple, yet invaluable, tool in the assessment of the morphology, mechanism and severity of the regurgitation. It provides important information about the impact of the lesion on the myocardium, adding prognostic data that influences patient management and treatment strategy. The use of advanced echocardiographic methods allows more precise quantification of regurgitation and estimation of subclinical myocardial dysfunction. Future studies that investigate the benefit of established and novel methods and link to clinical outcomes will improve our understanding of aortic regurgitation and enable improved clinical practice.

## Data Availability

All data presented in this manuscript are already published data that are publicly available and are cited throughout the document.
